# Using a spike-in experiment to evaluate analysis of LC-MS data

**DOI:** 10.1186/1477-5956-10-13

**Published:** 2012-02-27

**Authors:** Leepika Tuli, Tsung-Heng Tsai, Rency S Varghese, Jun Feng Xiao, Amrita Cheema, Habtom W Ressom

**Affiliations:** 1 Lombardi Comprehensive Cancer Center, Georgetown University, 4000 Reservoir Rd, Washington, DC, USA

**Keywords:** Difference detection, Label-free, Liquid chromatography-mass spectrometry (LC-MS), Spike-in peptide

## Abstract

**Background:**

Recent advances in liquid chromatography-mass spectrometry (LC-MS) technology have led to more effective approaches for measuring changes in peptide/protein abundances in biological samples. Label-free LC-MS methods have been used for extraction of quantitative information and for detection of differentially abundant peptides/proteins. However, difference detection by analysis of data derived from label-free LC-MS methods requires various preprocessing steps including filtering, baseline correction, peak detection, alignment, and normalization. Although several specialized tools have been developed to analyze LC-MS data, determining the most appropriate computational pipeline remains challenging partly due to lack of established gold standards.

**Results:**

The work in this paper is an initial study to develop a simple model with "presence" or "absence" condition using spike-in experiments and to be able to identify these "true differences" using available software tools. In addition to the preprocessing pipelines, choosing appropriate statistical tests and determining critical values are important. We observe that individual statistical tests could lead to different results due to different assumptions and employed metrics. It is therefore preferable to incorporate several statistical tests for either exploration or confirmation purpose.

**Conclusions:**

The LC-MS data from our spike-in experiment can be used for developing and optimizing LC-MS data preprocessing algorithms and to evaluate workflows implemented in existing software tools. Our current work is a stepping stone towards optimizing LC-MS data acquisition and testing the accuracy and validity of computational tools for difference detection in future studies that will be focused on spiking peptides of diverse physicochemical properties in different concentrations to better represent biomarker discovery of differentially abundant peptides/proteins.

## Background

Changes in the abundance of particular proteins/peptides or their modifications can influence the state of an organism. The primary focus of biomarker discovery studies in proteomics is to study changes in peptide/protein abundances that are generated in response to a perturbation, disease, morphogenesis, toxicity, or other cell stress in a given biological system [1-4]. Liquid chromatography-mass spectrometry (LC-MS) has been an indispensable tool for profiling small differences in expression level of peptides/proteins in complex biological medium [5-8]. This is due to the development of soft ionization techniques and tandem mass spectrometry, which makes it a sensitive tool for detecting and identifying peptides. Data-dependent acquisition (DDA) or information-dependent acquisition (IDA) of tandem mass spectra has allowed complex proteomes to be cataloged as well [9]. A typical LC-MS-based proteomics study includes sample preparation, separation of peptides/proteins on a single or multiple HPLC columns, ionization of chromatographic elutes by ESI source (electrospray ionization), detection of multiple charged peptides/proteins by mass spectrometry and subsequent data analysis.

Difference detection by LC-MS method can be achieved by either using isotope coded affinity tag method (ICAT) and other isotope labeling techniques such as iTRAQ, O^18 ^and N^15 ^[10-12] to encode relative abundance of peptides contained in two complex samples or using a label-free approach. Although isotope labeling methods have been widely used for both relative and absolute quantification of peptides abundance, several limitations remain including limited number of samples allowed, artifacts during labeling process, limited availability of isotope references, etc. [13,14]. Meanwhile, label-free methods have grown as an alternative for measuring protein/peptide abundances in biomarker discovery studies.

Label-free methods have been gaining a lot of attention for measuring differential protein expression as they are cost effective and yield a wide dynamic range. These methods do not require labeling of samples and involve simpler sample preparation protocols than labeling methods. Label-free difference detection methods have been used based on various features including spectral count [15], sequence coverage [16], peptide count [17], and precursor ion intensity [18-22]. The first three methods provide relative abundance information on the basis of MS/MS fragmentation. However, these methods tend to discard low abundant ions that are not typically selected at survey scan (MS1) level for MS/MS fragmentation. Alternatively, a direct comparison of precursor ions, where all ions at MS1 level are analyzed, can potentially capture low abundant ions. However, this approach requires that preprocessing steps such as peak detection, filtering, alignment, and normalization are appropriately handled with care.

**Figure 1:**
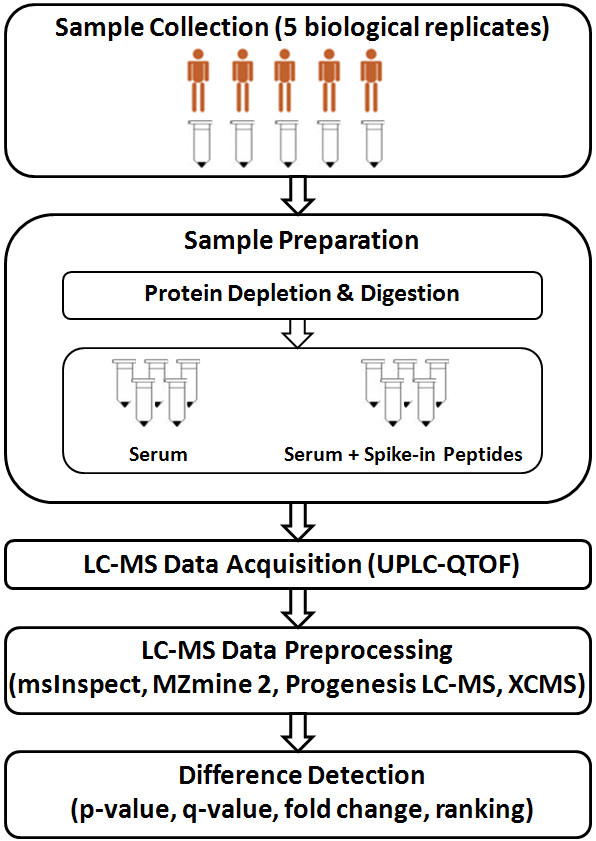
**Spike-in experiment to evaluate analysis of LC-MS data generated by a label-free LC-MS method**. The experiment design involves two groups of samples: (i) serum samples with spike-in peptides, and (ii) serum samples alone. LC-MS data were generated by UPLC-QTOF mass spectrometer. Four software tools (msInspect, MZmine 2, Progenesis LC-MS, and XCMS) were applied for LC-MS data analysis to evaluate workflows implemented.

An LC-MS run contains retention time information in a chromatogram, *m*/*z *value in MS spectrum, and relative ion abundance for each particular ion. MS signals of all ions across the whole chromatographic duration are formatted as a three-dimensional map that defines the LC-MS data. With an increasing interest in label-free quantification, several software tools have been made available for LC-MS data analysis including msInspect [23], MZmine 2 [24], Progenesis LC-MS (NonLinear Dynamics, United Kingdom) [25], and XCMS [26]. While all tools are involved in preprocessing LC-MS data at some level [27], they either have been optimized for certain platforms or have their own computational requirements on how the data should be processed. Each tool implements a different set of algorithms in its workflow characterized by its set of strengths and limitations. While some workflows perform well on data preprocessing steps, others focus primarily on statistical and machine learning methods for difference detection or visualization. The spike-in experiment presented in this paper allows us to evaluate such workflows. Figure 1 illustrates the experimental design that involves two groups of samples: (i) serum samples with spike-in peptides, and (ii) serum samples alone. LC-MS data were generated by QTOF mass spectrometer equipped with nano-ionization source and hyphenated with nano-Acquity UPLC system. Four software tools (msInspect, MZmine 2, Progenesis LC-MS, and XCMS) were applied for LC-MS data analysis to evaluate workflows implemented. The software tools were chosen for flexibility provided by their modular workflows to assess each pipeline explicitly and their ability to export information on extracted features for further comparison and statistical analysis.

## Results and discussion

**Figure 2:**
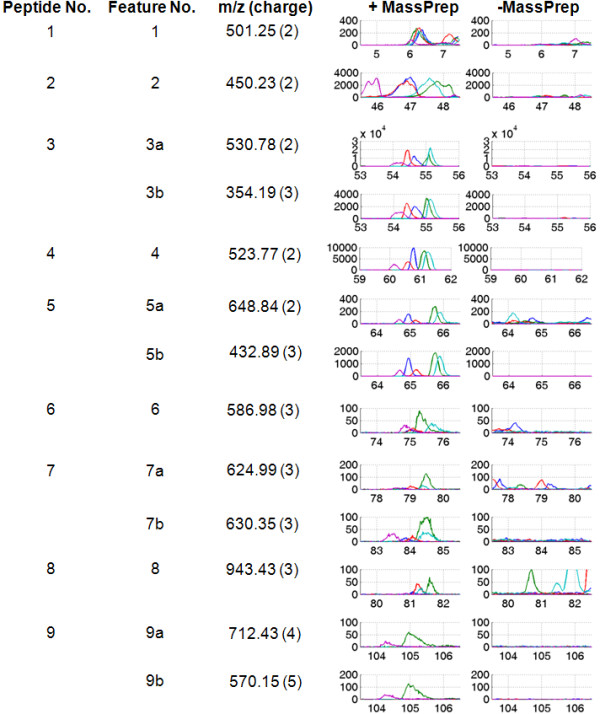
**Base peak chromatograms of the MassPrep peptides**. The chromatograms are zoomed into each of the 13 unique features whose *m*/*z *and retention time values match with those of the MassPrep peptides, in comparison of serum with spike-in peptides (+MassPrep) and serum alone (-MassPrep) groups.

Two groups of data were generated from five serum samples obtained from five healthy individuals. The first group of data was derived from the five serum samples mixed with known concentration of spike-in peptides. The second group of data was obtained from the five serum samples alone. In the first group, nine MassPrep peptides (designated as Peptides 1-9 in Table 1) were added prior to acquisition of LC-MS data (Figure 1). The data are available at http://omics.georgetown.edu/massprep.html. The raw LC-MS data were exported as .wiff files and converted to mzXML format using mzWiff (version 4.2.1) or msconvert from ProteoWizard (version 2.2) [28] before importing them into the software tools. Due to the compatibility issue between converters and analysis tools, data converted by mzWiff were analyzed by MZmine 2 while data converted by msconvert were analyzed by the other three tools. Figure 2 demonstrates the presence/absence contrast between the two groups with the base peak chromatograms of the spiked MassPrep peptides in *m*/*z *accuracy ± 50 ppm and retention time tolerance of 3 min. Parameters for each preprocessing pipelines were chosen to identify as many spike-in peptides as possible.

**Table 1:** The sequence information of the nine selected MassPrep peptides

**Peptide no**.	Component name	Molecular weight (g/mol)	pKa	Peptide sequence
1	RASG-1	1000.4938	9.34	RGDSPASSKP
2	Angiotensin frag 1-7	898.4661	7.35	DRVYIHP
3	Bradykinin	1059.5613	12.00	RPPGFSPFR
4	Angiotensin II	1045.5345	7.35	DRVYIHPF
5	Angiotensin I	1295.6775	7.51	DRVYIHPFHL
6	Renin substrate	1757.9253	7.61	DRVYIHPFHLLVYS
7	Enolase T35	1871.9604	7.34	WLTGPQLADLYHSLMK
8	Enolase T37	2827.2806	3.97	YPIVSIEDPFAEDDWEAWSHFFK
9	Melitin	2845.7381	12.06	GIGAVLKVLTTGLPALISWIKRKRQQ

At the feature detection level, all the 13 features can be detected in at least one of the four software tools. For difference detection at MS1 level, we calculate the *p*-value of *t*-test on each detected feature and apply multiple testing correction [29]. Features that satisfy the criterion of *q*-value < 0.05 and fold change (FC) > 10 are selected as significantly different between the two groups. Prior to this statistical analysis for difference detection, we retained only the features that were detected in at least two replicates in each group. Among the features detected by msInspect, 6,525 features were considered for statistical analysis, of which 2,099 had *q*-value < 0.05 and FC > 10. From 12,092 features obtained by MZmine 2, 539 features yielded *q*-value < 0.05 and FC > 10. From 8,415 features obtained by Progenesis LC-MS, 467 features had a *q*-value < 0.05 and FC > 10. Among 8,703 features from XCMS, 66 features had *q*-value < 0.05 and FC > 10. Table 2 presents the number of features selected from each of the four software tools following statistical analysis. The table presents the features that represent the MassPrep peptides and which resulted in significant changes (*q*-value < 0.05 and FC > 10) in comparing MS1 level LC-MS data from the two groups. Also, the table shows the ranking of the peptides on the basis of *q*-values and FC as well as the sensitivity and number of false positives of the results obtained in analyzing the data preprocessed by each of the four software tools.

**Table 2:** Performance comparison of msInspect, MZmine 2, Progenesis LC-MS, and XCMS.

msInspect (Number of selected features = 2099, sensitivity = 9/13, FP = 2090)				
**Feature No**.	**Peptide**	***m*/*z *(charge)**	***q*-value (rank)**	**FC (rank)**

1	RGDSPASSKP	501.25 (2)	0.0139 (38)	Inf (1)
2	DRVYIHP	450.23 (2)	0.0114 (1)	Inf (1)
3a	RPPGFSPFR	530.78 (2)	0.0164 (43)	90.5 (1911)
3b	RPPGFSPFR	354.19 (3)	0.0114 (1)	32.5 (1935)
4	DRVYIHPF	523.77 (2)	0.0166 (220)	47.8 (1921)
5a	DRVYIHPFHL	648.84 (2)	0.0120 (28)	Inf (1)
5b	DRVYIHPFHL	432.89 (3)	0.0126 (31)	Inf (1)
6	DRVYIHPFHLLVYS	586.98 (3)	0.0164 (43)	Inf (1)
7a	WLTGPQLADLYHSLMK	624.99 (3)		
7b	WLTGPQLADLYHSL(M)K	630.35 (3)	0.0164 (43)	Inf (1)
8	YPIVSIEDPFAEDDWEAWSHFFK	943.43 (3)		
9a	GIGAVLKVLTTGLPALISWIKRKRQQ	712.43 (4)		
9b	GIGAVLKVLTTGLPALISWIKRKRQQ	570.15 (5)		

MZmine 2 (Number of selected features = 539, sensitivity = 7/13, FP = 532)				

Feature No.	Peptide	*m*/*z *(charge)	*q*-value (rank)	FC (rank)

1	RGDSPASSKP	501.25 (2)	0.0278 (18)	40.0 (243)
2	DRVYIHP	450.23 (2)	0.0271 (1)	97.9 (152)
3a	RPPGFSPFR	530.78 (2)	0.0271 (1)	54.1 (202)
3b	RPPGFSPFR	354.19 (3)	0.0271 (1)	41.4 (238)
4	DRVYIHPF	523.77 (2)	0.0278 (18)	204.9 (107)
5a	DRVYIHPFHL	648.84 (2)	0.0399 (173)	11.2 (509)
5b	DRVYIHPFHL	432.89 (3)	0.0301 (53)	9636.3 (34)
6	DRVYIHPFHLLVYS	586.98 (3)		
7a	WLTGPQLADLYHSLMK	624.99 (3)		
7b	WLTGPQLADLYHSL(M)K	630.35 (3)		
8	YPIVSIEDPFAEDDWEAWSHFFK	943.43 (3)		
9a	GIGAVLKVLTTGLPALISWIKRKRQQ	712.43 (4)		
9b	GIGAVLKVLTTGLPALISWIKRKRQQ	570.15 (5)		

Progenesis LC-MS (Number of selected features = 467, sensitivity = 8/13, FP = 459)				

Feature No.	Peptide	*m/z *(charge)	*q*-value (rank)	FC (rank)

1	RGDSPASSKP	501.25 (2)	0.0308 (180)	73.0 (178)
2	DRVYIHP	450.23 (2)	0.0103 (1)	33.1 (240)
3a	RPPGFSPFR	530.78 (2)	0.0146 (4)	32.4 (243)
3b	RPPGFSPFR	354.19 (3)	0.0103 (1)	46.9 (210)
4	DRVYIHPF	523.77 (2)	0.0146 (4)	65.8 (184)
5a	DRVYIHPFHL	648.84 (2)		
5b	DRVYIHPFHL	432.89 (3)	0.0146 (4)	Inf (1)
6	DRVYIHPFHLLVYS	586.98 (3)		
7a	WLTGPQLADLYHSLMK	624.99 (3)		
7b	WLTGPQLADLYHSL(M)K	630.35 (3)	0.0222 (33)	50.6 (201)
8	YPIVSIEDPFAEDDWEAWSHFFK	943.43 (3)		
9a	GIGAVLKVLTTGLPALISWIKRKRQQ	712.43 (4)		
9b	GIGAVLKVLTTGLPALISWIKRKRQQ	570.15 (5)	0.0441 (365)	Inf (1)

XCMS (Number of selected features = 66, sensitivity = 7/13, FP = 59)				

Feature No.	Peptide	*m*/*z *(charge)	*q*-value (rank)	FC (rank)

1	RGDSPASSKP	501.25 (2)	0.0437 (3)	40.0 (8)
2	DRVYIHP	450.23 (2)	0.0224 (1)	25.3 (19)
3a	RPPGFSPFR	530.78 (2)	0.0437 (3)	52.2 (7)
3b	RPPGFSPFR	354.19 (3)	0.0437 (3)	38.0 (9)
4	DRVYIHPF	523.77 (2)	0.0437 (3)	137.8 (2)
5a	DRVYIHPFHL	648.84 (2)		
5b	DRVYIHPFHL	432.89 (3)	0.0437 (3)	488.4 (1)
6	DRVYIHPFHLLVYS	586.98 (3)		
7a	WLTGPQLADLYHSLMK	624.99 (3)		
7b	WLTGPQLADLYHSL(M)K	630.35 (3)	0.0491 (66)	17.9 (27)
8	YPIVSIEDPFAEDDWEAWSHFFK	943.43 (3)		
9a	GIGAVLKVLTTGLPALISWIKRKRQQ	712.43 (4)		
9b	GIGAVLKVLTTGLPALISWIKRKRQQ	570.15 (5)		

The comparison is based on q-value and fold-change (FC) of spike-in peptides in comparing spike-in serum samples versus serum samples. Ranking is provided on q-values and FC. The number of differentially abundant features, sensitivity, and false positives (FP) are calculated based on the criterion of q-value < 0.05 and FC > 10

As shown in Table 2, six features (1, 2, 3a, 3b, 4, and 5b) were selected as differentially abundant (*q*-value < 0.05, FC > 10) by all four software tools while three features (7a, 8, and 9a) were selected by none of the software tools. The remaining four features (5a, 6, 7b, and 9b) were selected by at least one software tool.

While msInspect and Progenesis LC-MS yielded relatively better sensitivity than XCMS and MZmine 2, the latter two got fewer false positives than the former two. However, it should be emphasized that by selecting more stringent parameters for msInspect and Progenesis LC-MS and focusing only on the high abundance peptides, comparable (or even fewer) number of false positives could be achieved. Our evaluation of software tools in this study highlights the challenges of using these tools for identifying low intensity features and boosting of sensitivity. This becomes more critical in biomarker discovery studies, where identifying low abundance features is very relevant. In general, without any information regarding features at MS1 level, it would be difficult to select parameters for each software tool properly. Using the MS/MS information of the MassPrep peptides and examining the associated MS1 profiles, the study design provides some guidelines for better extracting features of interests. Missing value is another critical issue for difference detection, particularly affecting low abundance peptides considerably [30]. Features detected in only a subset of the replicates (e.g., Features 7a, 8, and 9a as depicted in Figure 2) may fail to pass statistical tests and not be selected as differentially abundant.

In addition to using parametric statistical test (*q*-values) and fold change (FC), difference detection results were also confirmed using the Wilcoxon rank-sum test (*p *< 0.05), which is a non-parametric statistical hypothesis test. Although it is always preferable to check which assumption holds true prior to using a specific statistical test, we chose to examine the results by applying both parametric and non-parametric tests due to small sample size and a large number of features involved in this study. A summary of the results is presented in Table 3. We observed that the combination of fold change, parametric test, and non-parametric test significantly reduces the number of false positives without significantly affecting the sensitivity.

**Table 3:** Summary of difference detection results by combining different statistical tests.

	msInspect	MZmine 2	Progenesis	XCMS
Total number of features detected	31168 (12)	12271 (12)	9267 (9)	21486 (13)

Number of features used for statistical analysis	6525 (9)	12092 (9)	8415 (9)	8703 (10)

*t*-test (*q *< 0.05)	4824 (9)	3505 (7)	4465 (9)	1896 (7)
Wilcoxon rank-sum test (*p *< 0.05)	603 (8)	1318 (6)	1584 (8)	812 (8)
*t*-test (*q *< 0.05) + Wilcoxon (*p *< 0.05)	603 (8)	967 (6)	1379 (8)	672 (7)
*t*-test (*q *< 0.05) + FC (> 10)	2099 (9)	539 (7)	467 (8)	66 (7)
*t*-test (*q *< 0.05) + Wilcoxon (*p *< 0.05) + FC (> 10)	388 (8)	323 (6)	238 (7)	55 (7)

The total number of features detected and those satisfying different criteria, i.e., *t*-test with multiple hypothesis testing (*q*-value < 0.05), Wilcoxon sum-rank test (*p*-value < 0.05), and fold change (FC > 10). The plus symbol denotes the combination of different criteria. Only features present in at least two replicates in each group were used for statistical analysis. The numbers in parenthesis indicate the number of MassPrep features identified in each category. We found six features (Features 1, 2, 3a, 3b, 4, and 5b) identified as differentially abundant by each of the four software tools in each category

## Conclusions

We present a spike-in experiment to evaluate the performance of four software tools in detecting "true differences" in peptide abundance between two datasets acquired using a label-free LC-MS method. The performance of each tool is assessed by their ability to detect spike-in peptides as true differences between two groups on the basis of MS1-level ion abundance measurements. We observe that selection of appropriate parameters for feature detection in using each software tool is very important. Modular workflows are desired to assess the parameters for each pipeline explicitly. In addition to the preprocessing pipelines, choosing appropriate statistical tests and determining suitable significance level are not trivial problems. We observe that individual statistical tests could lead to different results due to different assumption and employed metrics. It is therefore preferable to incorporate several statistical tests for either exploration or confirmation purpose. The LC-MS data from our spike-in experiment can be used to assess the statistical power of different testing methods.

While Progenesis LC-MS supports a complete pipeline for label-free proteomics and provides information on conflicting peptides identified for the same feature, certain parameters for peak grouping are not available for tuning/optimization. Whereas the other three software tools provide the flexibility for software parameterization and extended analysis, they lack an identification module. Each step in the preprocessing workflow is important as highlighted by the analysis results. While developing a flawless workflow seems difficult, it would be extremely beneficial if preprocessing steps from different tools could be unified into a single workflow. At present, most software tools do not provide such flexibility which prevents the possibility to borrow strength with each other.

As shown in Table 3, all the 13 expected features that represent the nine MassPrep peptides can be detected by at least one software tool. Of these, between seven and nine features were selected as significantly different between the two groups (Table 2). At peptide level, Peptide 8 was never selected, whereas Peptides 7 and 9 were selected as differentially abundant at charge +3 and +5, respectively. For Peptide 9, Feature 9b with charge +5 was observed to be the most prominent ion. The identity of this feature was confirmed based on MS/MS data collected on its 3rd isotopic peak. A low resolution setting on Q1 quadropole allowed all isotopes associated with a given precursor to be transmitted through the quadropole, thereby generating a 3rd isotopic peak for Feature 9b. Although the IDA experiment is set to monitor peaks with charge states +2 to +4, sometimes when the software cannot determine the charge state, it will consider the peak as *unknown *and that peak will be submitted for MS/MS if its intensity is above a pre-specified intensity threshold. We believe that Feature 9b was selected for MS/MS analysis, because it was tagged *unknown*. Similarly for Peptide 7, a modified peptide sequence (Feature 7b, methionine oxidation at residue #15 that has a mass difference of 16 Da) was observed to be prominent. On the other hand, while Peptide 8 was easily detected and identified in absence of serum, no such matching feature was observed consistently in the spiked-in serum group. A possible explanation is ion suppression from co-eluting interferences in serum sample during ESI. While Features 7a, 8 and 9a may appear to be non-informative from the point of software evaluation, they highlight an often forgotten issue with LC-MS data, where the inability to detect an analyte does not necessarily imply its absence from the sample. Since Feature 8 (representing Peptide 8) only had a low ion intensity and was detected in only a subset of the samples in presence of serum, further optimization at sample preparation and LC-MS gradient level is needed. Differential mass spectrometry combined with targeted MS/MS analysis of only identified differences may save both computation time and human effort compared to shotgun proteomics approaches. The work in this paper is an initial study to develop a simple model with "presence" or "absence" condition using spike-in experiments and to identify these "true differences" using available software tools. The LC-MS data from our spike-in experiment can be used for developing and optimizing LC-MS data preprocessing algorithms and to evaluate workflows implemented in existing software tools. Current work as a stepping stone can help optimize the LC-MS data acquisition and test the accuracy and validity of computational tools for difference detection in future studies that will be focused on spiking peptides of diverse physicochemical properties in different concentrations to better represent the biomarker discovery of differentially abundant peptides/proteins.

## Methods

### Sample collection and materials

Serum samples were obtained from five healthy individuals after centrifugation of blood samples at 1,000 g for 15 min. Samples of serum were stored at -80°C and allowed to thaw on ice at room temperature prior to analysis. Acetonitrile and Acetone were purchased from Fisher Scientific. Tris (2-carboxyethyl) phosphine (TCEP), methyl methanethiosulfonate (MMTS), Trypsin (human pancreas) were purchased from sigma Aldrich.

### Protein depletion and digestion

Abundant proteins such as albumin obscure separation and detection of low abundant proteins in serum. It is common to deplete serum of high abundant proteins such that low abundant proteins can be easily detected. For our study, we depleted 60 μL of human serum of Immunoglobulin G (IgG) and Albumin using Aurum serum protein mini columns as described in BioRad protocol [31]. Depleted serum was collected as the unbound elute by centrifuging the column at 10,000 g. Protein concentration was determined by Bradford assay. After acetone precipitation, 100 μg of depleted serum was set for trypsin digestion. Samples were reduced with 50 mM tris (2-carboxyethyl) phosphine (TCEP) at 60°C for 60 min. After cooling to room temperature, the sample was alkylated using 0.2 mM methyl methanethiosulfonate (MMTS) at 37°C for 30 min. Trypsin was added (protein:enzyme ratio of 50:1) into protein solution and incubated at 37°C for 3 h. After that, trypsin was added one more time into the previous digestion mixture allowed for overnight incubation at 37°C. The digested samples were further cleaned using Reverse phase Macrospin column (NestGroup, Inc.), speedvac dried at 40°C, and reconstituted in mobile phase A solvent (0.1% formic acid in H_2_O, 2% ACN) prior to injection.

### LC-MS data acquisition

Two groups of data were generated from five serum samples obtained from five healthy individuals. The first group of data was derived from the five serum samples mixed with known concentration of spike-in peptides while the second group of data was obtained from the five serum samples alone. Both groups of data were acquired by the same LC-MS method. In the first group, nine MassPrep peptides (designated as Peptides 1-9 in Table 1) were added prior to acquisition of LC-MS data (Figure 1). The MassPrep peptide mixture is a careful selection of nine peptides with a wide range of polarities, and isoelectric points (pI) (Table 1). These peptides were preferred as they come with a predictable retention behavior and elution order as well as span a broad chromatographic time range. The concentration of the MassPrep peptides (1 pmol/μL) was selected and injection volume was 1 μL. Different concentrations of MassPrep peptides (0.05, 0.1 and 0.5 pmole/μL) were also evaluated (data not shown). MassPrep peptides mixture with concentration of 1 pmole/μL showed the minimal intensity that would not swamp the MS signals of serum peptides in LC-MS acquisition. This amount of peptides was also used in other spike-in studies [32]. There are other available LC-MS datasets with spike-in peptides. Listgarten et al. [33] simulated a contrast between the "presence" and "absence" conditions of spike-in peptides similar to our effort, but they generated the ground truth by processing the MS1 data rather than referring to the MS/MS identification results. Another dataset by Mueller et al. [34] was designed for a scenario of different concentrations of six proteins. However, their standard solutions were mixed with isolated human serum peptides that may not fully reflect the complexity of serum samples. In addition, no biological replicates were considered in the two datasets.

Chromatographic separation was performed on Waters NanoACQUITY system using BEH C18 column: 75 *μ*m × 150 mm, 1.7 *μ*m particle size that was equilibrated with 99% mobile phase A (0.1% formic acid in H_2_O, 2% ACN) and 1% mobile phase B (0.1% formic acid in acetonitrile, 2% H_2_O). The protein digest was preloaded on a nanoACQUITY UPLC Symmetry C18 trap column (180 *μ*m × 20 mm) before separation. The column and the autosampler were maintained at a temperature of 40°C and 4°C respectively. A 180 min gradient elution (in a 240 min run) was performed at 300 nL/min flow rate as following: maintain 99% mobile phase A for the first 20 min, change organic solvent composition to 80% mobile phase B in 180 min, then increase to 99% B in 15 min and equilibrated to 99% A and for 25 min. The nanoACQUITY UPLC was connected to QTOF-MS (AB Sciex QSTAR Elite) through nano-electrospray ionization source. The automated system ensured reproducible loading of samples using LC-autosampler.

The mass spectrometer was externally calibrated by two ions of *m*/*z *879.9723 and *m*/*z *110.0713 using porcine rennin substrate tetradecapeptide (1 *μ*M in 30:70:0.1 (v/v/v) acetonitrile:water:formic acid). After calibration, resolution was obtained above 10,000 in FWHM, mass accuracy less than 10 ppm and ion intensity above 2,000. MS settings were as follows: Ionspray voltage (IS) 2,300, Interface heater temperature (IHT) 160°C for the positive ionization mode, with a mass tolerance set to 100 mDa. Analyst (version 2.0) was used for data acquisition and LC-MS operations. A full mass scan (MS1) was obtained with a resolution between 9,000-12,000. One scan cycle of seven seconds included one MS scan from *m*/*z *350-2000 and five MS/MS scans that are triggered by information dependent acquisition (IDA) to acquire for MS/MS information on five most intense precursor ions ranging from *m*/*z *50 to 2000.

Information dependent acquisition enables the "on-the-fly" acquisition of MS/MS spectra during a chromatogram run, in which an MS survey scan is used to generate peak list of all ions (precursor ions) and only ions that meet the defined criteria, such as threshold intensity, *m*/*z *range and charge state, etc., are subjected for MS/MS. The QTOF-MS with IDA setup enables acquisition of both TOF MS1- survey scan as well as MS/MS- product ions scan. This cycle is repeated throughout the duration of acquisition. IDA experiment monitors five most intense peaks that exceed 25 counts in intensity with charge state +2 to +4 and mass tolerance 100 ppm. Former target ions were excluded for 30 s. Automatic collision energy and MS/MS accumulation were also applied during MS/MS acquisition. Ions of MassPrep peptides were added to an inclusion list under IDA mode to trigger the acquisition of their MS/MS spectra. Based on previous runs, the MassPrep peptides detected in TOF MS survey scan could not be selected for MS/MS analysis due to their low signal intensities and presence of co-eluting serum peptides. However, by including MassPrep peptide ions into the inclusion list in IDA acquisition, MS/MS information of spike-in peptides could be obtained for the purpose of later identification. For each batch of samples, the inclusion list was prepared based on one injection of MassPrep peptides run at the beginning of a batch.

It is important to note that while spectral comparison was primarily done at MS1 level, MS/MS data were not needed for comparison purposes. However, MS/MS information was used for assigning identification to significant features, later in the analysis. Peptides identified with a > 90% confidence level were retained on the list. This file was imported into the Inclusion tab of the IDA method in Analyst software. Both time window of ± 1 min and theoretical *m*/*z *value of each peptide were put on the inclusion list. During a batch run each sample was followed with four wash cycles to minimize sample carryover. A 100 fmol *E.coli *beta-galactosidase digest was run at the beginning and end of a batch to monitor the performance of LC-MS acquisition.

The data were searched against the International Protein Index (IPI) human (version 3.6) database using Paragon Algorithm in ProteinPilot™ software (version 3.0). Fixed modification was set to carboxymethyl (C) and variable modification to oxidation of methionine (M). A cutoff value of 95% confidence or 1% false discovery rate (FDR) was used only when searching raw data against IPI database via Paragon algorithm. For Progenesis LC-MS software, a MASCOT search was done against a custom MassPrep database (MPDB), containing MassPrep peptides and human protein sequences with MassPrep sequence similarity. Both groups (i.e., with and without spike-in peptides) were searched against MPDB. Compiling a custom MPDB was necessary as certain MassPrep peptides were not present in human database, thus reducing the database search time considerably. MPDB was used in evaluating the analysis results from Progenesis LC-MS and it was observed that significant features identified as MassPrep peptides were assigned to with a high MASCOT score.

### Analytical tools

We evaluated four software tools: msInspect, MZmine 2, Progenesis LC-MS, and XCMS. All tools perform the common steps including peak detection, peak grouping, alignment, and difference detection. In addition, Progenesis LC-MS allows peptide identification by analysis of tandem MS (MS/MS) data. All tools except XCMS provide solutions to identify charge states and isotopic patterns. Detected features were further filtered to retain those present in at least two replicates in each group for statistical analysis.

The main workflow in msInspect (version 2.3, build 599) is composed of three modules called findpeptides, filter, and peptidearray [23]. It starts with peak detection followed by deisotoping and charge state recognition. Based on demands of users, the detected features can be further filtered by intensity, *m*/*z *range, or scan range. The features are then aligned and grouped across samples by the peptidearray module. Alignment is based on either splines or quantile regression and is only applied to features of identical charge states. The selected parameters are summarized as follows: FeatureStrategyPeakClusters and walkSmoothed options were selected for findpeptides module; minIntensity = 15 and minScans = 10 were selected for filter module; quantile option, minPeaks = 2, minCharge = 1, scanWindow = 125, alignmztolerance = 0.025, and massWindow = 0.1 were selected for peptidearray module.

A workflow composed of peak detection, peak list deisotoping, alignment, peak row filtering and gap filling is carried out in MZmine 2 [24]. The peak detection is performed by linking intense ions with similar *m*/*z *across successive scans to build chromatograms. The deconvolution algorithm is then applied to each chromatogram to recognize individual chromatographic peaks. With specified *m*/*z *and retention time tolerances, the alignment can be carried out with either linear or nonlinear correction. The parameters for each preprocessing step in MZmine 2 are summarized as follows. For each LC-MS run, noise level of 2.5, minimum intensity of 5, retention time span of 10 s, and *m*/*z *tolerance of 0.1 Da were selected for peak detection and filtering purpose; maximum charge of 6, retention time tolerance of 3 s, and *m*/*z *tolerance of 0.03 Da were selected for deisotoping. Feature alignment across runs was based on the parameters: *m*/*z *tolerance of 0.05 Da and retention time tolerance of 1.5 min. Gap filling for missing value imputation was then applied to the detected features.

Progenesis LC-MS generates an aggregate run containing the peptide ions from all analyzed runs. Semi-supervised alignment is achieved by choosing a reference run and aligning all runs to it based on landmark vectors. The landmark vector is composed of landmark points of retention times and *m*/*z *values that enable alignment of all LC-MS runs. In Progenesis LC-MS, the landmark vector can be generated either automatically by the software for all LC-MS runs based on detected paired features (details are not provided by Progenesis) or can be selected manually by looking at each LC-MS map. For our study we placed 10 manual points consistently present in all LC-MS runs. After alignment, the isotopic patterns are identified and irrelevant features are then filtered out by selecting a retention time range, charge states and number of isotopes. Even though the LC run was 240 min, for our analysis we observed most of the peptides were eluted before 150 min (during the gradient). The LC-MS map is therefore filtered to retain features within 150 min of retention time. In spite of peptides coming out earlier, the LC was run was longer to segregate serum peptides from junk coming off at > 200 min with high organic. Once filtered the data are normalized by calculating quantitative abundance ratio to the reference run. Normalization is based on the assumption that most proteins do not change, and so the quantitative abundance ratio should be close to 1. This is followed by aggregating runs for difference detection and statistical analysis.

XCMS provides a series of LC-MS data preprocessing algorithms implemented in R language [26] and is helpful to assess performance at each preprocessing step. The peak detection is performed on overlaid extracted ion chromatograms by a pattern matching approach, with kernel of Gaussian or second derivative of Gaussian. After peak detection, peaks that fall within a pre-defined range are grouped together and better grouped peaks are identified as "well-behaved" peaks for alignment purpose. Alignment is preceded by applying medium filter and local regressor. Since XCMS was developed for metabolomics study, several issues related to proteomics study such as charge state selection and isotopic pattern identification are not addressed. The parameters for each preprocessing step in XCMS are summarized as follows. For peak detection, the matched filter algorithm with parameters fwhm = 30, snthresh = 10, profstep = 0.1, and an intensity threshold of 5 was used. Retention time correction was performed two times with peak grouping in between with bw = 30, mzwid = 0.1 after first retention time correction and bw = 15, mzwid = 0.1 after the second retention time correction.

## Abbreviations

ACN: Acetonitrile; DDA: Data-dependent acquisition; ESI: Electrospray ionization; fmol: Femtomole; ICAT: Isotope-coded affinity tag; IDA: Information dependent acquisition; iTRAQ: Isobaric tag for relative and absolute quantitation; LC: Liquid chromatography; Da: Dalton (molecular mass); MS: Mass spectrometry; MS/MS: Tandem mass spectrometry; *m*/*z*: Mass-to-charge ratio; pmol: Picomole; S/N: Signal-to-noise ratio; QTOF: Quadruple time of flight; UPLC: Ultra performance liquid chromatography.

## Competing interests

The authors declare that they have no competing interests.

## Authors' contributions

TL carried out the experiment, participated in the statistical analysis and drafted the manuscript. THT performed the statistical analysis and drafted the manuscript. RSV participated in the statistical analysis. JFX and AC provided expertise in experimental design and interpretation of analytical results and drafted the manuscript. HWR conceived of the study, designed the experiment and drafted the manuscript. All authors read and approved the final manuscript.
